# Introduction to the special issue in honor of Juan Jose Dolado

**DOI:** 10.1007/s13209-022-00262-y

**Published:** 2022-05-10

**Authors:** Laura Mayoral, Evi Pappa

**Affiliations:** 1 Institute for Economic Analysis (CSIC), Barcelona, Spain; 2 Universidad Carlos III de Madrid, Getafe, Spain

- *“Have you ever spotted a pink elephant? It’s really hard to find a pink elephant because elephants are grey.”*

This is a typical phrase in Juanjo’s lectures. But he would have probably never imagined that we, who know him well, actually do spot the pink elephant in the classroom. It is the lecturer.

Juanjo is the incarnation of an outlier, a personality that lies at an abnormal distance from other researchers in a random sample of academics in Spain, in Europe, and worldwide. It is difficult to summarize the merit and life of such an extraordinary scholar in a few paragraphs, so we hope he will forgive us if we fall short.

Juanjo Dolado, JD, would have had the best initial conditions for becoming a disc jockey, all he had to have done was switch his initials to DJ. He started flirting with this idea early in life, but then tradition led him to study to the Universidad Complutense de Madrid, where he completed his B.A. in Economics in 1977 and in Mathematics in 1979.

Madrid was great at the time, but could not be compared to London, which was really the place to be! The year 1979 marked the beginning of several trends in music. Electropop was one of them, quickly reaching the top of the British charts. It was time to move there. In 1978 Juanjo and his classmate Rafael Repullo were granted the Bank of Spain scholarship for graduate studies abroad and were admitted to the MSc in Econometrics and Mathematical Economics at the LSE. Two notable classmates in their cohort were John Moore (who did not know any economics and was not afraid of asking) and Dilip Mookherjee (who instead knew everything). The teachers in the program were also outstanding: George Akerlof, Partha Dasgupta, Amartya Sen, Douglas Gale, Chris Pissarides, Steve Nickell, David Hendry, Denis Sargan, and Ken Binmore, among others. But, at least in Juanjo’s mind, this was nothing compared to the number of concerts taking place in London and its surroundings at the time. Between September 1979 and June 1980, while in the first year of the program, Juanjo attended, according to his own recollection, 65 concerts, more than twice in a week. The list of rock stars was also impressive: The Clash, Sex Pistols, Police, Slits, Chic, Jab Jab, Elvis Costello, Richard Hell, John Cooper, UFO, Liar, Osmonds and Frank Zappa, among others.

Rafa and Juanjo shared a room in a nice street in Chelsea, the best thing about it being that it was close to a good record store. On the way to the tube station, Juanjo bought a single almost every day, to break the monotony of the long hours of study. They also shared the record player: 1 h of classical music (Rafa) and 1 h of pop (Juanjo). As the English of both flat mates was not great, they gave some of the most remarkable seminar presentations in the history of the LSE. In a memorable one, Juanjo discussed the consequences of “changing tastes” (which led to inconsistent intertemporal choices), pronouncing ‘’tastes’’ as “testes” (meaning testicles in English). The Brits were polite, but the seminar stuck with them for a while.

It was prophetic that one of the top hits of 1981 was *“Tainted Love”,* by *Soft Cell*. The opening of the song describes very precisely Juanjo’s feelings during that period. *“Sometimes I feel, I've got to run away, I've got to get away…”* Contrary to Rafa, who stayed on for the PhD, Juanjo went back to Madrid to become an employee at the Bank of Spain, despite having admission and funding from the LSE.

During these golden years at the Bank, he was offered to give a number of lectures to executives, and this kept him entertained. 1983 was both a revelation and a decisive year in his life. While teaching an Econometrics course, he met Samuel Bentolila, who was finishing his undergraduate studies. Juanjo asked Samuel to send him his thesis, he read it right away and gave him comments the very next day. Samuel was amazed. They have been friends ever since. At the European meeting of the Econometric Society in Pisa, he met a couple of young academics, Olympia Bover and Manuel Arellano, another long-lasting pillar in Juanjo’s life.

1983 was ending and Juanjo kept a copy of Blondie’s *“The Hunter”* on his desk, a concept album based on the theme of *"searching, hunting, or pursuing one's own Mt. Everest."* With this soundtrack, he identified young and beautiful Carmen Contreras as his Everest. She was brought to his apartment by one of his flat mates, who was also attending his course. Carmen was planning to informally attend the course as well and has been by his side ever since.

How could Juanjo have escaped his deserved academic destiny in this network of young and promising Spanish academics? In 1985 both Olympia and Manuel took jobs at Oxford. The instigator of this move was Steve Nickell, Olympia's PhD advisor at the LSE and Manuel's mentor, who had also moved from LSE to Oxford the year prior. Juanjo and Carmen followed them that same year, and Juanjo started a PhD at Oxford with David F. Hendry and Steve Nickell as advisors.

From then on, there were only *“Happy Mondays,” “Gang of Four”—*or six, if one includes Anindya Banerjee and Antonio Villar, also PhD students at Oxford at the time—Oxford *“Culture Clubs”* and outings*.* Life was *“Simply Red” and* Juanjo had time to *“Talk Talk”* for the following 5 years. He built his econometrics and economics skills in the post-punk New Wave era. He had been extremely productive. Life at Oxford *“Spined Him Round (Like a Record)"* and, before he even realized it, he became a father: In December 1987, Laura Dolado Contreras was born at the John Radcliffe Hospital and spent the first moments of her life surrounded by millions of records, loose papers with mathematical proofs and the tender love of her parents. The thesis entitled "Modelling Employment, Inventories and Wages in U.K. Manufacturing Sector: A Cointegration Approach" was defended in 1988 and, after that, Juanjo became a lecturer at Oxford for two more years.

The UK chapter ended in 1990 when the Dolado-Contreras family moved back to Madrid. Juanjo came back to Spain, as an *“Englishman in Madrid”*.^1^ But good news was waiting again around the corner. Ana Dolado Contreras arrived in early February 1991 to keep Juanjo happily busy. Olympia and Manuel also returned to Madrid that year and, thanks to Carmen’s efforts, they settled into a house neighboring theirs in Chamartín. It is still inexplicable why Juanjo chose to live so close to the Bernabeu stadium, the source of all evil for all Atletico de Madrid fans.

Another five years passed. By then, Juanjo had become a CEPR research fellow, a member of the Economic Policy Panel and Associate Editor of the Revista Española de Economía. He had published 25 academic articles in Spanish and 31 articles in English, together with a number of policy reports. During those years he published some of his most influential econometrics papers: "The Power of Cointegration Tests," (1992) with N.R. Ericsson and J.J.M. Kremers, “Co-integration, error correction, and the econometric analysis of non-stationary data,” (1993) with A. Banerjee, J.W. Galbraith, and D. Hendry, “Cointegration and unit roots,” (1990) with T. Jenkinson and S. Sosvilla, and “Making Wald tests work for cointegrated VAR systems,” (1996) with H. Lütkepohl.

This academic success, however, failed to impress some of the Bank’s highly ranked staff. So Juanjo left the Bank for CEMFI with a leave of absence in 1996. This year was another benchmark year for many reasons: Most importantly Atletico Madrid finally won the League after a dry spell of 19 years. Secondly, Juanjo found at CEMFI a friendly environment, accompanied by his best friends Manuel, Samuel and Rafa. After two years there, Rafa made him an offer to stay permanently but fate had a different destiny for Juanjo. The offer from CEMFI never materialized. Instead, an offer from UC3M arrived, with Juan Urrutia pulling the strings; both CEMFI and the Bank lost out, Madrid’s Carlos III gaining an excellent academic in 1998. As *Jewel* was singing at that time, *"Foolish Games"* were over and Juanjo was meant for UC3M.

While all these changes undoubtedly constituted a headache for Juanjo at the time, they were a stroke of luck for one of the authors here, who was looking for a thesis supervisor at the precise moment that Juanjo landed his new destination. She could not have wished for a better mentor. Juanjo’s arrival at Carlos III was also a breath of fresh air for everybody there. And Getafe’s atmosphere probably also inspired Juanjo, who remained faithful to his relentless productivity (41 research articles and 6 book contributions), besides being head of the Department of Economics (1998–2001), writing music reviews for Expansion y RockdeLux and recommending films and records left and right. He also began then his fruitful collaboration with Jesús Gonzalo and started a tradition: publishing in Econometrica shortly after joining that University (see Dolado et al. 2002a, b). There were certainly difficult moments as well, possibly none as hard as having to prematurely say farewell to his beloved friends and co-authors Ramón María Dolores and Francesc Mármol.

Ever since his Bank of Spain and CEMFI times, Juanjo dedicated a good part of his research to issues related to the analysis of the labor market, with a special interest in the Spanish one.^2^ This established him as one of the leading labor economists in Europe. He wrote seminal articles on the topic, such as “Labour flexibility and wages: lessons from Spain,” (1994) with S. Bentolila; "The Growth Effects of Migration in the Host Country," (1994) with A. Ichino and A. Goria; and "The Economic Impact of Minimum Wages in Europe" with F. Kramarz, A. Manning, S. Machin and C. Teulings. In his first years in UC3M, together with another long-lasting co-author, Juan Francisco Jimeno (and continuing his collaboration with Samuel and other co-authors), Juanjo published more than 15 articles in top journals examining the dynamics of labor markets in Spain and in Europe. See Dolado et al. (1997), Boldrin et al. (1999). Dolado and Jimeno (1997), Dolado et al. (2002), Bentolila et al. (2008), Bentolila et al. (2012), and more recently, Bentolila et al. (2020).

In line with Juanjo’s interests, this special issue includes papers studying the Spanish Labor market. In “*Lost in Recessions: Youth Employment and Earnings in Spain*”, *Samuel Bentolila, Florentino Felgueroso, Marcel Jansen and Juan Francisco Jimeno* talk to the heart of Juanjo’s concerns: youth unemployment in Spain, an issue in which Juanjo has maintained a strong interest throughout his career (see Dolado, Felgueroso and Jimeno, 2000 and Dolado, Felgueroso and Jansen, 2013). As was reflected in the panel discussion and in Juanjo’s speech during the conference organized in his honor, youth unemployment is a major challenge for the Spanish economy and more research and effort should be dedicated to address it. In this paper the authors estimate the effects for college-educated workers of entering the job market in a recession. In “*The internship contracts in Spain: a stepping stone or a hurdle towards job stability?*” *Lucía Gorjón and Sara de la Rica* evaluate the impact of the internship contract (IC), which was originally designed to promote an appropriate transition for young people with higher education from their formative years to their working lives. “*Temping Fates in Spain: Hours and Employment during the ‘Great Recession’ and Covid-19*” by *Cristina Lafuente Martinez, Raul Santaeulalia-Llopis and Ludo Visschers* studies the behavior of the number of hours worked in Spain in the most recent recessions while “*So different yet so alike: micro and macro labour market outcomes in Germany and Spain*”, by *Maia Guell, Cristina Lafuente, Manuel Sanchez and Helene Turon* documents differences in unstable labor market spells across these two countries. These last two papers are very closely related to Juanjo’s research agenda, see Dolado, García-Serrano and Jimeno (2002) and Booth et al. (2002). In “*Higher education decisions and macroeconomic conditions at age eighteen*”, *Jennifer Graves and Zoe Kuehn* study how macroeconomic conditions experienced at age eighteen affect a variety of decisions in post-secondary and tertiary education. Juanjo has also examined the factors determining educational performance, see Dolado and Morales (2009).

Another significant area of his research has been dedicated to gender differences in labor market outcomes. In the seminal paper of Dolado et al. (2004), the authors analyze the segregation of labor markets by gender; De la Rica et al. (2008) analyze gender wage gaps and their determinants, while De la Rica et al. (2013) propose a model of self-fulfilling prophecies in which statistical discrimination results in both wage and housework time differences across ex-ante identical individuals, except for their gender; De la Rica et al. (2015), study the gender wage and performance gap in Spain; and more recently, gender differences are investigated in the particular episode of the Great Recession in Dolado et al. (2020). In “*Work and children in Spain: Challenges and opportunities for equality between men and women*”, *Claudia Hupkau and Jennifer Graves* highlight the fact that the gender inequalities Juanjo and his co-authors have identified in previous research are further aggravated among women with children.

In related work, Dolado et al. (2012) analyze the distribution of research fields by gender in the top-50 Economics departments, concluding that women are unevenly distributed across the fields. In “*Gender Distribution across Topics in Top 5 Economics Journals: A Machine Learning Approach*”, J. Ignacio Conde-Ruíz, Juan-José Ganuza, Manu García and Luis A. Puch revisit this subject by examining the distribution of topics by gender across all articles published in top-five journals in the period 2002–2019, reaching similar conclusions.

A new chapter in Juanjo’s life started in January 2014 when he decided it was about time to get “*a room with a view”* in Florence and moved to the European University Institute. Going from a huge university as Carlos III de Madrid to a small villa with 12 professors was a big challenge for Juanjo at the beginning, also because he did not speak any Italian. While it was so easy to talk with Amado from the central cafeteria in Getafe about Atletico de Madrid’s matches, Juanjo quickly realized that Loredana’s (the lady preparing coffees in Villa San Paolo) knowledge of the intricacies of Atletico de Madrid was severely lacking. It was not easy for the faculty either. No one was used to daily visits to discuss the royal family gossip from “Hola” extracts, new movies being released, football scores or Champion League highlights. But above all no one was prepared to cope with the avalanche of CDs and the newcomer’s insistence of having them played immediately.

Juanjo soon realized that EUI’s treasure is its students, so they quickly became the recipients of his energy and enthusiasm. First the younger crowds in Villa San Paolo and, later, in Villa La Fonte. In this special issue we have three contributions of his students and disciples at the EUI. In “*From He-Cession to She-Stimulus? The Labor Market Impact of Fiscal Policy across Gender*”, *Alica Ida Bonk and Laure Simon* analyze the differential effects of fiscal policy on female labor market outcomes. The authors find that the effects of fiscal policy shocks on labor market outcomes depend on the type of public expenditure. Many of the results of this research were discussed in the benches of “Mago Balducci” bar in the neighborhood of “Le Cure,” or in car drives from downtown Florence to Fiesole. Some of these rides were tough. Juanjo can get carried away when discussing economics and music. He pines for people to appreciate the songs he enjoys and so, while driving, he typically switches frantically from one CD to the next, playing half a song from each, making you wonder if you will survive the ride. Could this be the reason why “*Automation and Sectoral Reallocation*” of *Dennis Hutschenreiter, Tommaso Santini and Eugenia Vella* talks about automation? Regardless, we are all hopeful that AI will someday help Juanjo change the CDs in his car. Eugenia and her coauthors show that in theory the reduction in manufacturing employment can be offset by the increase in service employment and for that reason the industrial robot adoption might not affect aggregate employment as it happened in Germany. “Primary elections and electoral outcomes: Evidence from the Spanish Socialist Party” by *Ricardo Ciacci, Jorge García Hombrados, Laura Gismera, Antonio Nuñez Partido* is also related to Juanjo’s years in the EUI, the contributor Riccardo Ciacci being Juanjo’s PhD student in Florence. This paper studies the causal impact of holding primary elections on electoral outcomes.

During his exile in Florence, the jury of the King Jaime I Prize for Economics decided to award the 2015 Prize to Juanjo, for his contributions in two different areas of economics. First, for his work on the methodology of cointegration and other techniques in Time Series econometrics. Second, for his contribution to the study of the labor market, particularly its duality and its effects on the dynamics of unemployment. This special issue reflects his influence in both fields.

One of the areas in which Juanjo has made outstanding contributions recently is in the study of factor models (see Chen et al. 2014, 2021). In “*Dynamic factor models: Does the specification matter?*”, Esther Ruiz, Pilar Poncela and Karen Miranda empirically analyze the behavior of alternative estimators of dynamic factor models (DFM) under various sources of misspecification. In particular, they focus on factor estimation and in-sample and out-of sample forecasting. *“Moment tests of independent components*”, by Enrique Sentana, Dante Amengual and Gabriele Fiorentini, focuses on the identification of shocks in structural VAR models. More specifically, it proposes specification tests that check the normality as well as potential cross-sectional dependence of the shocks.

Something that we hope is clear by now is Juanjo’s out of the ordinary, if not to say extraordinary, character. In their article “*A Tale of three cities: Climate heterogeneity*” Jesús Gonzalo and María Dolores Gadea explore a surprising hypothesis: that maybe the climate of the cities in which Juanjo has lived can explain his sociability, outgoing character and his capacity for hard and diverse work. To explore this conjecture, they analyze the climate and warming patterns in three key cities in Juanjo’s life: his current city of residence (Madrid), his birth town (Zaragoza) and the city where he did his PhD studies (Oxford). They conclude that although the three cities are experiencing warming, the patterns are quite different. Unfortunately, they don’t reach definitive conclusions on the impact of warming patterns on Juanjo’s personality, which hopefully will be clarified in future research.

In January 2019, the belltower of Puerta del Sol welcomed both the new year and Juanjo’s return to Madrid and Carlos III. Everything had changed yet remained the same in Getafe. A simple walk with Juanjo from one building to another would still take you about 30 min, since Juanjo talks to the gardener, the porter, the bar tender and some students on the way. And at lunch you’d be hopeful that it’s time to eat, but would lose him yet again to another table and then another one, busier than a groom at his own wedding. But endless corridor talks aside, despite his incessant musings on the latest music and Netflix series, one day Juanjo will skip into your office, happy as a child, telling you that his old tradition has been upheld, yet again—the tradition of, shortly after arriving at UC3M, he will be publishing a paper in *“Econometrica”* (Chen et al., 2021). Juanjo, the true *“Rocketman.”*

And to finish as we started, we will go back to the initial quote. Juanjo resembles an elephant from another perspective: he has a prodigious memory. Apart from papers and their exact methodology and number and name of co-authors and journals and issues they have published in, he can remember completely random stuff, like the name of the boyfriend of a PhD student he met in Florence seven years ago, the name of the wife and kids of any bar tenders he’s been served by, the year someone got tenured, got promoted or her thesis title. His memory is amazing! So, we hope he will forgive us for leaving something out from this extensive report on his academic and personal life. Usually, the introductory comments of a volume are just a few paragraphs long. We went over this limit, as it was really tough to fit a “pink elephant” into such a small space.

And given the unusualness of our introductory comments, we would also like to finish in keeping by paraphrasing “It’s an elephant world”, by Bruce Cockburn:

Great thrills are in store for us all,

*while the world is on such a slant,*

*though he's a bit funky*

*the students love his trunk!*

*He's a hit, he is exuberant,*

*but they want to retire our elephant.*

*If you should meet him, please give us a call.*

*We do not want him to retire at all!*

*You're bound to see him*

*at *the* classroom or a record shop.*

*Even though some people can't.*

*Get used to believing in elephants*

*Do not call the guard*

*He's playing another card*

*He bungled this seminar for sure*

*He blew it in style*

*But he's just a child!*

We love you, Juanjo.

Laura and Evi and co.

## Footnotes

1 We borrow this term from Jesús Gonzalo’s talk in the conference in Juanjo’s honor, Madrid 13th–14th December 2021. See https://sites.google.com/view/conference-in-honor-of-dolado/program.

2 *Mondays In The Sun* (*Los Lunes Al Sol*, 2002) is a Spanish drama directed by F. León de Aranoa, starring Javier Bardem and Luis Tosar. The film depicts the degrading effects of unemployment on a group of men left jobless by the closure of the shipyards in Vigo, Galicia. Juanjo likes films as much as he likes music, and draws inspiration from both. Although he started working on the Spanish labor market before the movie was released, we are sure that the film inspired him to write more papers on the issue.
